# Learning from diagnostic errors to improve patient safety when GPs work in or alongside emergency departments: incorporating realist methodology into patient safety incident report analysis

**DOI:** 10.1186/s12873-021-00537-w

**Published:** 2021-11-18

**Authors:** Alison Cooper, Andrew Carson-Stevens, Matthew Cooke, Peter Hibbert, Thomas Hughes, Faris Hussain, Aloysius Siriwardena, Helen Snooks, Liam J. Donaldson, Adrian Edwards

**Affiliations:** 1 Division of Population Medicine, School of Medicine, Cardiff University, Cardiff, UK; 2 University of Warwick, Coventry, UK; 3 Macquarie University, Sydney, Australia; 4 John Radcliffe Hospital, Oxford, UK; 5 University of Lincoln, Lincoln, UK; 6 Swansea University, Swansea, UK; 7 London School of Hygiene and Tropical Medicine, London, UK

**Keywords:** Patient safety, Diagnostic error, General practitioners, Emergency department

## Abstract

**Background:**

Increasing demand on emergency healthcare systems has prompted introduction of new healthcare service models including the provision of GP services in or alongside emergency departments. In England this led to a policy proposal and £100million (US$130million) of funding for all emergency departments to have co-located GP services. However, there is a lack of evidence for whether such service models are effective and safe. We examined diagnostic errors reported in patient safety incident reports to develop theories to explain how and why they occurred to inform potential priority areas for improvement and inform qualitative data collection at case study sites to further refine the theories.

**Methods:**

We used a mixed-methods design using exploratory descriptive analysis to identify the most frequent and harmful sources of diagnostic error and thematic analysis, incorporating realist methodology to refine theories from an earlier rapid realist review, to describe how and why the events occurred and could be mitigated, to inform improvement recommendations. We used two UK data sources: Coroners’ reports to prevent future deaths (30.7.13–14.08.18) and National Reporting and Learning System (NRLS) patient safety incident reports (03.01.05–30.11.15).

**Results:**

Nine Coroners’ reports (from 1347 community and hospital reports, 2013–2018) and 217 NRLS reports (from 13 million, 2005–2015) were identified describing diagnostic error related to GP services in or alongside emergency departments. Initial theories to describe potential priority areas for improvement included: difficulty identifying appropriate patients for the GP service; under-investigation and misinterpretation of diagnostic tests; and inadequate communication and referral pathways between the emergency and GP services. High-risk presentations included: musculoskeletal injury, chest pain, headache, calf pain and sick children.

**Conclusion:**

Initial theories include the following topics as potential priority areas for improvement interventions and evaluation to minimise the risk of diagnostic errors when GPs work in or alongside emergency departments: a standardised initial assessment with streaming guidance based on local service provision; clinical decision support for high-risk conditions; and standardised computer systems, communication and referral pathways between emergency and GP services. These theories require refinement and testing with qualitative data collection from case study (hospital) sites.

## Background

Increasing demand on emergency healthcare systems has prompted the introduction of new service models, including provision of General Practitioner (GP) services in or alongside emergency departments [1]. In England this led to a policy proposal and £100million (US$130million) of funding for all emergency departments to have co-located GP services [2]. The aim of this initiative was to reduce waiting times and overcrowding and therefore improve overall patient care and safety [3, 4], however, there is a lack of evidence about potential patient safety risks associated with these service models and how these could be mitigated [5, 6].

Estimates for the number of patients presenting to emergency departments with primary care type problems that can be dealt with by GPs vary from 10 to 43%, depending on definitions and population groups [7–13]. There are several different models of GP service provision associated with emergency departments: INSIDE the emergency department, either *integrated* with the emergency medicine service or in a separate *parallel* service; or OUTSIDE the emergency department, either *on or off site* [14]. These models may function on a spectrum from being closer to an emergency medicine service or to usual primary care service provision. As well as GPs, the service may also include nurse practitioners and other primary care healthcare professionals [14].

Emergency departments are high-risk settings for diagnostic errors that may result in significant patient harm [15, 16]. The World Health Organization has also identified diagnostic errors in primary care as a high-priority problem [17]. GPs traditionally have different diagnostic approaches to emergency medicine clinicians. They rely less on acute investigations [8, 18], and may have different approaches or cognitive biases from working in lower risk settings [19]. There is little research evidence to guide decisions about how GP service models in or alongside emergency departments can be most effective and safe, and how to minimise the risk of diagnostic errors [5, 6, 20, 21].

Patient safety incident reports can be aggregated to generate data summaries describing the most frequent and harmful incident types while thematic analysis of individual report texts can be used to explore how and why such incidents may occur and identify contributing factors that can be targeted to mitigate future events [22]. Studies of incident reports using this approach have been used to develop improvement recommendations to prevent safety-related hospital deaths [23], the treatment of children in primary care [24], and diagnostic errors in undifferentiated emergency department attendances [25].

This work was part of a wider NIHR funded realist evaluation evaluating the effectiveness of GPs working in or alongside emergency departments [26], conducted following a rapid realist review of the effectiveness of GPs working in or alongside emergency departments [6]. Realist methodology includes learning from ‘nuggets of information’ to explain what works, for whom, how and in what circumstances to generate *theories* described as context-mechanism-outcome configurations (CMOs) [27, 28]. We aimed to analyse two UK data sources of patient safety incident reports to: characterise the nature of diagnostic errors related to these GP services; refine theories developed from the rapid realist review to explain how and why these incidents occurred to inform qualitative data collection and subsequent theory testing at hospital case study sites; and inform improvement recommendations.

## Methods

We conducted a four-stage sequential exploratory mixed-methods analysis of two UK national data sources: Coroners’ reports to prevent future deaths, and patient safety incident reports from the National Learning and Reporting System (NRLS). This notably incorporated realist principles as follows:

1. **Familiarisation of report content and application of codes** from the PISA frameworks to create coded summaries of report narratives [22]*.*
2. **Generation of data summaries** using exploratory descriptive statistics to describe the frequency and burden (harm) of incident types and key relationships with contributory factors.
3. **Interpretation of themes and learning** through a thematic analysis of reports aggregated by common characteristics from step 2. We used realist methodology to infer why incidents may have occurred to identify additional contributing factors which were not explicit from report narratives read in isolation. We identified mechanisms (M) that explained how or why contexts (C) related to outcomes (O) to develop theories described as context-mechanism-outcome configurations (CMOs), definitions in Table 1 [27, 28].
4. As per realist methodology, an **additional stage of stakeholder expert feedback** was added to validate findings.

**Table 1:** Realist definitions [27, 28]

**Realist definitions**	
Context (C)	Pre-existing conditions which influence the success or failure of different interventions or programmes
Mechanism (M)	Characteristics of the intervention and people’s reaction to it; how it influences their reasoning
Outcome (O)	Intended and unintended results of the intervention as a result of a mechanism operating within a context
Initial rough theory	An early theory, informed by available evidence, about how, why, for whom, and in what circumstances the intervention is thought to work described as a context-mechanism-outcome (CMO) configuration
Refined theory	An initial theory that has been refined using primary or secondary evidence

### Data sources

#### Coroners’ reports to prevent future deaths

According to the Coroners and Justice Act 2009, Coroners have a statutory duty to make reports to a person, organisation, local authority, government department or agency if they believe that action should be taken to prevent future deaths [29]. All reports and responses must be sent to the Chief Coroner and most cases are summarised and published on the Courts and Tribunals Judiciary publicly available website [30].

#### National Reporting and learning System (NRLS) patient safety incident reports

The NRLS is a database of over 18 million patient safety incident reports, usually reported by staff, from healthcare organisations in England and Wales. A patient safety incident is defined as, “any unintended or unexpected incident that could have harmed or did harm a patient during healthcare delivery” [31]. Reporting began voluntarily in 2003 but, since 2010, it has been mandatory to report any incident that resulted in severe patient harm or death. Since the inception of the NRLS, reporting arrangements have included batch returns via local risk management systems, and more recently in England, by direct notification to the Care Quality Commission (an independent regulator of all health and social care services in England). Reports contain anonymised, structured information about location, patient demographics, and the reporter’s perception of harm severity, complemented by unstructured free-text descriptions of the incident, potential contributory factors, and planned actions to prevent reoccurrence.

### Sampling strategy

Pilot work was conducted in January 2017 to identify a sample of NRLS reports regarding GP services in or alongside emergency departments, but most were irrelevant referring to the GP as part of the patient’s journey rather than the GP service. Since the sample of identified Coroners’ reports all described diagnostic error, although infrequent, we included these recognised patient safety incident data as they record the highest level of patient harm and may be especially informative.

#### Coroners’ reports to prevent future deaths

We reviewed all reports available in the ‘Community health care and emergency services related deaths’, ‘Hospital Death’ and ‘Child Death’ categories (2013–2018) on the Courts and Tribunals Judiciary website in August 2018. Reports were selected if they were related to GP service provision in or alongside emergency departments (inclusion and exclusion criteria shown in Table 2).

**Table 2:** Inclusion and exclusion criteria

**Inclusion criteria** • Reports describing diagnostic errors related to GP services in or alongside emergency departments **Exclusion criteria** • Reports involving community ‘in-hours’ or ‘out-of-hours’ GP service provision not occurring at the same geographical location either within or alongside emergency departments • Diagnostic errors occurring during usual emergency department service provision	

#### National Reporting and learning System (NRLS) patient safety incident reports

**Fig. 1:**
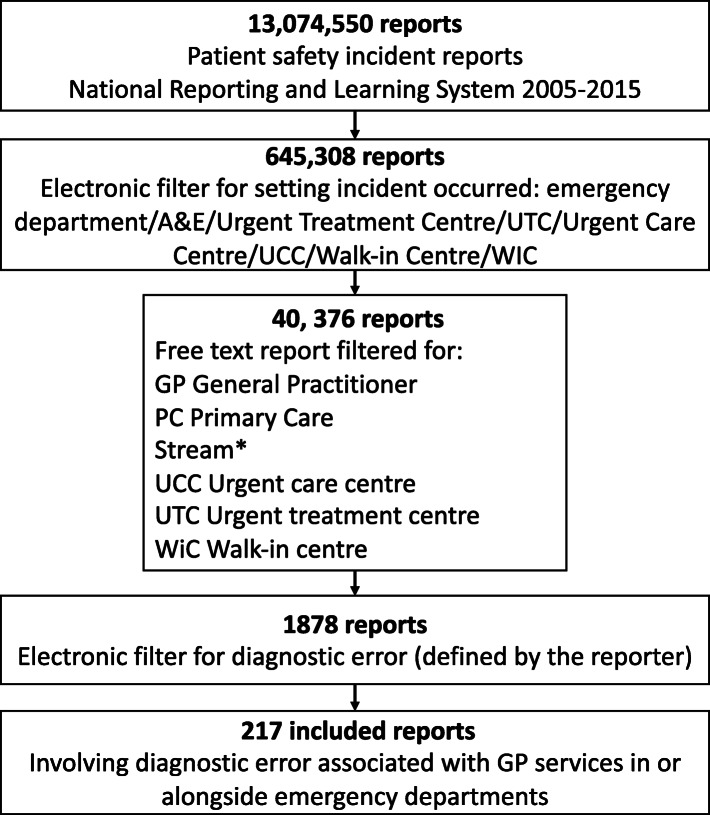
National Reporting and Learning System patient safety incident reports, search strategy and results

NRLS reports were available from 03/01/05–30/11/15, stored on a secure computer platform at Cardiff University. We filtered reports with structured electronic variables (pre-specified by the reporter or their organisation before submission to the NRLS) for emergency and urgent care settings (PD05); then the free-text with primary care terms to identify reports regarding GP services in these settings; then using structured variables for diagnostic error as defined by the reporter (IN05), Fig. 1. Searches were conducted July–September 2018. After this three-stage filtering process, we read the reports to determine if they were related to GP service provision in or alongside emergency departments (Table 2).

### Data analysis

#### Familiarisation and data coding

We coded reports from both datasets using the multi-axial PISA classification system based on the recursive model for incident analysis and aligned to the four major classes of information advocated by the World Health Organisation International Classification for Patient Safety [22]. This involved reading the free text component of each report and coding information in relation to: the *primary* safety incident that was reported to have directly affected patient care (e.g. diagnostic error); the *chain of incidents* leading up to the safety incident (e.g. misinterpretation of X Ray); other independent *contributory factors* (e.g. unusual patient presentation); and reported patient *harm outcomes* with harm severity classified from the free text report according to WHO International Classification for Patient Safety definitions [32]. We classified diagnostic errors according to the Society to Improve Diagnosis in Medicine (Table 3) [33]. The primary coder was an experienced GP and patient safety qualitative researcher (AC). A medical student (FH) double coded the NRLS reports (259/1878, 14%) and took part in coding meetings to clarify the recursive model approach, the nature of the codes and discuss complex cases. FH was working on a parallel study analysing NRLS reports describing diagnostic error associated with usual emergency department care [25]. The Cohen’s kappa showed acceptable moderate agreement (0.51) between coders.

**Table 3:** Diagnostic error definitions [33]

Diagnostic error definitions	
Diagnostic error	The failure to (a) establish an accurate and timely explanation of the patient’s health problem(s) or (b) communicate that explanation to the patient.
Wrong diagnosis	Occurs, for example, if a patient truly having a heart attack is told their pain is from gastro-oesophageal reflux.
Delayed diagnosis	The diagnosis should have been made earlier.
Missed diagnosis	Medical complaints never explained, or more specific complaints never accurately diagnosed.

#### Generation of data summaries

﻿We exported the codes into Microsoft Excel for mac (version 16.35) and used pivot tables to undertake an *exploratory descriptive analysis* to assess the nature of the diagnostic error and the most frequent and harmful presenting conditions from both datasets, the associated chain of incidents, and other contributory factors. We summarised the most commonly identified causes and potential interventions in a driver diagram [34]. This is a quality improvement tool to summarise priority areas for change and to map potential interventions.

#### Thematic analysis and realist theory development

We then used *thematic analysis* to identify and describe recurring themes within and between datasets (not captured by the quantitative data) that could be targeted to mitigate future similar incidents, incorporating realist methodology to infer why incidents may have occurred, to whom, in what circumstances and why. We used the initial rough theories developed from the rapid realist review as a template for mapping supporting data on the Microsoft Excel spreadsheet [6]. If the data gave additional information to explain how and why the event may have occurred, we incorporated this information into the CMO configuration to refine the theory. New information, not included in the initial rough theories from the review, was used to develop new initial theories.

#### Stakeholder feedback

We presented findings to the wider ‘GPs in EDs’ team for feedback: including patient safety experts, patient representatives, primary care and emergency department clinicians (TH, PH, ACS, FD, ME, PA, JH, BE) at a study meeting in November 2018. Feedback was strongly supportive. We also carried out literature searches to determine whether existing interventions or initiatives for promoting patient safety had been described in each area and aligned these with the driver diagram intervention areas. When available, the strength of each intervention was graded using the US Department of Veterans Affairs classification, where the strongest designs are permanent and physical rather than temporary and procedural [35].

## Results

### Coroners’ reports to prevent future deaths

We screened 1347 Coroners’ reports to prevent future deaths classified as “Community health care and emergency services” and “Hospital deaths” over the five-year period. From these we identified nine cases which included diagnostic errors related to GP service provision in or alongside emergency departments (summarised in Table 4). No new cases were identified in the “Child Death” category (with some duplication of cases found in the other sections).

**Table 4:** Summary of Coroners’ reports to prevent future deaths related to GP service provision in or alongside emergency departments (9 reports identified from 1347 reports, 2013–2018)

Report number	Presenting symptom	Initial diagnosis	Actual diagnosis	Summary of report	Key learning from reports
1. Wrong diagnosis	Calf pain	Muscular injury	Deep vein thrombosis (DVT)	A 47-year-old woman presented to the urgent care centre with calf pain. She had a strong family history of DVT but this was not elicited in the history and she was diagnosed with muscular pain. She later died from a pulmonary embolism.	*“The A&E expert gave evidence that patients presenting to an urgent care centre, walk in centre or out of hours are a much* ***higher risk group than those who present to their own GP surgery****. As a consequence, there* ***must be clinically agreed protocols*** *that at the front end of any facility that receives undifferentiated patients that manage this higher risk population.* ***Patients that present with certain high risk conditions such as chest pain, shortness of breath or calf pain must be directed to a facility that can exclude serious illness and this is usually the nearest A&E.”***
2. Wrong diagnosis	Calf pain	Muscular injury	Deep vein thrombosis (DVT)	A man presented to a walk in centre with calf pain following a driving holiday in France. There was no calf swelling or tenderness and he was diagnosed with a musculoskeletal injury. He was then seen by his own GP a further 3 times but the walk in centre records were not available. He later died of a pulmonary embolism.	*“****Records*** *of the August appointment (to the walk-in centre)* ***were not available****.”*
3. Wrong diagnosis	Shortness of breath	Not documented	Pulmonary embolism (PE)	A 44-year-old man presented to A&E and was streamed to the GP. He died from a pulmonary embolism two days later.	*“Mr (),* ***died of a pulmonary embolism*** *having been* ***diverted from accident and emergency assessment*** *2 days prior to his death. This meant that* ***further tests, which could have led to an earlier diagnosis for his condition were not done****. No 111 referral information was available to ‘Front door’ or the ED (emergency department).”*
4. Wrong diagnosis	Chest pain	Non-cardiac chest pain	Adult Cardiac Death Syndrome	A 30-year-old woman presented to the ambulance service with chest pain, normal examination and ECG. She chose to see her GP who thought the pain was non-cardiac, she died a few hours later at home.	*“Mrs Y, aged 30 with a family history of heart disease, was seen by ambulance staff with chest pain, and examination and ECG were reported as normal.* ***The GP had not considered the possibility of Sudden Adult Death Syndrome”***
5. Wrong diagnosis	Chest pain	Gastritis	Loeys-Dietz Syndrome (thoracic aneurysm)	A 42-year-old woman with chest pain was seen by an ambulance, had a normal ECG and chose to see her GP for review. She was seen by the local GP and referred to A&E for further investigation. She was streamed to the GP in A&E who referred her back to A&E where she was assessed, treated for gastritis and discharged with no further investigations. The patient’s presenting history of the same pain as her previous aortic dissection and the initial GP referring letter was lost in transfer. She died a few days later.	*“Crucially,* ***the only piece of the patient’s presenting history which wasn’t passed on (to the ED doctor from the local GP) was that the pain that she was feeling was the same pain which she had felt back in 2011 when she suffered her previous aortic dissection****. Had he been aware of this piece of information, his evidence was that he would have ordered a CT scan.”*
6. Wrong diagnosis	Head injury	Not documented	Intracranial haemorrhage	A man presented to an urgent care centre following a head injury and again the following day with headache and vomiting. No CT was done. He collapsed and died the next day.	*“****Patients undergoing haemodialysis or significant uraemia are at risk of haemorrhage*** *and this is not commonly known within the medical profession or referred to in relevant NICE guidelines.”*
7. Wrong diagnosis	Head injury	Not documented	Extradural haematoma	A 10-year-old boy presented to A&E following a head injury and was streamed to the urgent GP clinic and discharged. He was seen at home by a paramedic the following day and not brought to hospital. He collapsed the next day whilst waiting to be seen in the GP surgery. He underwent neurosurgery but died a few days later.	*“The consultant from the department told me, during the course of his evidence, that* ***it would be good practice for all suspected head injuries to be referred to the A&E team.”***
8. Delayed diagnosis	Unclear	n/a	Sepsis	A patient presented to the emergency department and was booked into the urgent care centre. He was not triaged for over 45 min by which time his condition had deteriorated.	*“****Staffing levels in the emergency department were not sufficient*** *to be able to follow national or any local policy on treating suspected sepsis.”*
9. Missed diagnosis	Cough	Chest infection	Pneumonia	A 9-month-old baby presented to a walk-in centre 3 times over 3 months with a cough. She was then seen twice by nurse practitioners at her own surgery with the same complaint who could not recall having access to information about the walk in centre visits and did not refer the patient to the GP. She died the following month from bronchopneumonia.	*“There* ***appeared to be no guidelines or triggers for when a practice nurse (practitioner) should refer a patient be seen by a doctor.”***

Seven of these reports described a wrong diagnosis with a lack of referral for investigation on initial presentation. Three main conditions were identified: veno-thrombotic events presenting with calf pain or shortness of breath (*n* = 3); cardiac death with a presentation of chest pain (*n* = 2); and intracranial haemorrhage following a head injury (*n* = 2). Another report described delayed initial assessment and diagnosis for a patient, which was felt to have contributed towards his death by sepsis. A further report described a missed diagnosis where a lack of communication about recurrent attendances from a walk-in centre was thought to have contributed to the death of a baby with pneumonia.

Root cause analysis and expert opinions were often detailed in the reports, giving understanding of the factors that may have contributed to the diagnostic errors. Patient characteristics included those presenting with rare conditions, for example Loeys-Dietz syndrome (thoracic aortic aneurysm), or others presenting with an atypical pattern of signs and symptoms, including no leg swelling in a patient presenting with a deep vein thrombosis or a young female with chest pain. The possibility of cognitive biases affecting the clinical reasoning of GPs who may usually work in community settings, with a lower probability of serious disease, was raised in one expert opinion. Organisational factors that may have contributed towards diagnostic errors included: lack of clear streaming guidance for patients presenting with high-risk conditions; unclear referral pathways for patients sent in for further investigation by their local GP; and communication barriers between primary and secondary care.

### National Reporting and learning System reports

Over the ten-year period, 1878 reports were identified in the filtered sample and screened. Irrelevant and duplicate reports were excluded resulting in an included sample of 217 reports describing diagnostic errors with learning related to GP service provision in or alongside emergency departments (see Fig. 1).

The reports were generally brief, with limited information about contributory events, and most did not describe the patient harm outcome resulting from the diagnostic errors (*n* = 188). In those reports where harm could be ascertained, 11 reports described mild or moderate patient harm, 12 described severe harm and six described events leading to death. Three of the six reports describing a death involved patients presenting with headaches. From the nature of the serious diagnoses involved (Table 5), for those reports without harm descriptions, patient harm appears likely.

**Table 5:** Presenting conditions involved in diagnostic errors described in incident reports related to GP service provision in or alongside emergency departments (National Reporting and Learning System reports; 217/13million 2005–2015)

Presenting complaint	Number of NRLS reports	Examples of conditions involved (not always stated in the report)
Musculoskeletal injury	114	114 fractures 7 Hip and 6 Spinal fractures
Chest pain	18	15 Acute Coronary Syndrome
Unwell child	15	7 sick infants requiring resuscitation level care
Headache	14	6 Head injury 5 Subarachnoid haemorrhage 2 Brain tumour
Abdominal pain	9	3 Appendicitis 1 Ischaemic bowel
Shortness of breath	6	1 Acute asthma 1 Pneumothorax 1 Respiratory failure 1 Stridor
Limb pain – no trauma	4	2 Deep vein thrombosis 1 Ischaemic foot
Collapse	4	1 Cardiac arrest
Back pain	4	1 Pulmonary embolism 1 Abdominal Aortic Aneurysm 1 Spinal cord compression
Limb weakness	2	2 Stroke
Eye injury	2	1 Missed foreign body in eye
Rash	2	1 Measles
Other	13	1 Testicular torsion 1 Ectopic pregnancy 1 Anaphylaxis
Not documented	10	1 Pneumothorax 1 Trauma case
Total	217	

Most reports described patients leaving the emergency department with a wrong diagnosis (*n* = 144). These were largely due to errors in clinical decision-making: mis-interpretation of X-Rays later picked up by radiological reporting systems (*n* = 87), or under-investigation of key symptoms (*n* = 59). Other reports described a delayed diagnosis within the emergency department or which was identified at a later date (*n* = 71). Over half of these involved an inadequate triage or streaming process (*n* = 42). Others described inadequate specialist referral pathways from community or emergency department GP services (*n* = 21), or inadequate assessment and investigation. In this sample there were no reports of missed diagnoses (Society to Improve Diagnosis in Medicine definition, Table 2) [33].

### Initial theories developed from both data sources

**Fig. 2:**
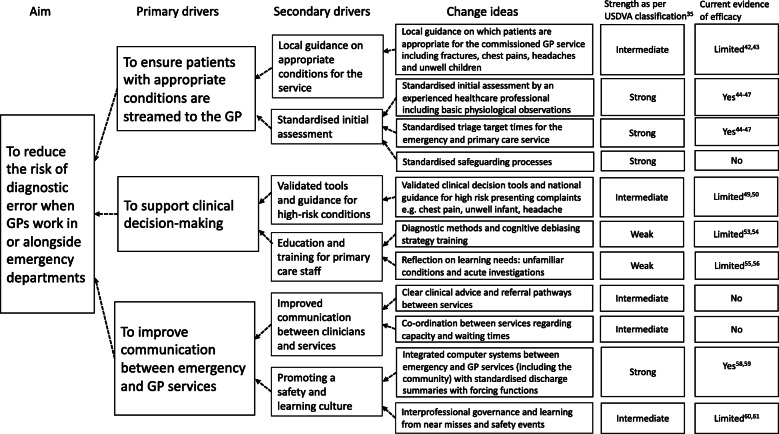
Driver diagram to show key areas to reduce the risk of diagnostic errors when GP services are located in or alongside emergency departments

We grouped contributory events that led to the diagnostic errors into three themes: difficulty with triage and streaming processes; errors in clinical decision-making including under-investigation and mis-interpretation of results; and organisational factors including inadequate referral pathways and communication between services. These are summarised in the driver diagram, along with interventions that could mitigate future events (see Fig. 2). Theories refined and developed from these data are described as context-mechanism-outcome configurations.

### Difficulty with triage and streaming processes

**Table:** 

If patients presenting to the emergency department (C) are assessed for streaming ***but the streaming nurse is unclear which patients are appropriate (due to unclear guidance or inexperience) (M)*** ***or the initial assessment is inadequate (limited history or lack of basic physiological observations) (M)*** then higher risk patients may be streamed to the GP service (O)	

*Bold text indicates how this theory was refined from the initial theory in the rapid realist review [6]

One Coroner’s preventing future death report and 29 NRLS reports highlighted the difficulty in identifying which patients were appropriate for the GP service. These included 10 patients presenting with chest pain and seven unwell children. A delay in the initial assessment for those streamed to the GP service (*n* = 14), inadequate assessment including basic observations (*n* = 13), and a lack of understanding of which patients were appropriate for the GP service (*n* = 7) were all described as contributing to events.

“A [>65 year old man] presented at [time] with ***dizziness*** and feeling like he was going to collapse. **No vital signs recorded by GP streamer. Waited 1 hour** to be seen in urgent care centre - when assessed by nurse practitioner heart rate 24 and BP unrecordable. **Transferred to resus**.”

### Errors in clinical decision-making

**Table:** 

If patients present to the emergency department with a ***condition not usually dealt with in primary care*** (C) and are seen by a GP who may have inadequate knowledge or skillset for the condition (M) the patient may be at ***risk of a mis-management*** (O)	

*This was a new theory generated from these data

Clinicians’ cognitive biases and the effect of working in a potentially higher risk environment on diagnostic thinking patterns were not documented. Six Coroners’ preventing future death reports and 59 NRLS reports described a lack of referral for urgent investigation. The conditions involved included: 27 fractures (four spinal and three hip fractures), 10 patients presenting with a headache (four following a head injury, four with confirmed subarachnoid haemorrhage), five patients with a deep vein thrombosis, four patients with acute coronary syndrome and four unwell infants.

Some NRLS reports described GPs taking on emergency clinician roles, interpreting acute investigations and managing patients with conditions not usually dealt with in community general practice. Most of these incidents were identified by radiological reporting systems, diagnosing fractures at a later date (*n* = 87). Others highlighted a lack of knowledge of child safeguarding protocols (*n* = 5) or the clinician’s inadequate skillset.

“Patient **attended the A&E department**, however **seen by the GP placed in the department** who examined the patient, ordered an x-ray and then **applied a plaster and discharged the patient** to fracture clinic. On reviewing the fracture, it was found to be a **comminuted fracture of the radial head**. This type of fracture would have **necessitated an orthopaedic review** as they benefit from early surgery. On reviewing the clinical notes **no mention of an orthopaedic review** was mentioned. It seems that the **practitioner concerned does not have the necessary experience to evaluate this type of injury** (this is not a primary care type of patient) and should not have managed the patient in the first place.”

### Inadequate referral pathways and communication between services

**Table:** 

If there is poor communication between the GP service and the emergency department service (C) because of a lack of awareness about capacity (M) **a lack of awareness that investigations have already been requested** (M) **or inadequate referral pathways** (M) then patient assessment and treatment may be delayed (O)	

*Bold text indicates how this theory was refined from the initial theory in the rapid realist review [6]

Inadequate specialist referral pathways, from the community or emergency department GP services, were described to contribute towards diagnostic errors in one Coroner’s preventing future death and 21 NRLS reports. Miscommunication between services about capacity and incompatible computer systems between the emergency department and GP service causing patients to get ‘lost’ in the system, ***in some cases leading to delays in treatment*** were described in another five NRLS reports. Under-staffing was described to contribute towards delayed assessment and diagnosis in one Coroner’s and one NRLS report. Another Coroner’s report highlighted a case where a lack of communication with community primary care regarding attendances to a walk-in centre possibly contributed towards the death of a baby.

“An infant [<2 years] **seen by primary care doctor**, discharged from PCC at [time 1] and **sent back to A&E**. Identified by primary care doctor in letter as unwell, laboured breathing and requiring further assessment and treatment however **not referred to paediatricians or informed A&E**. As a result the child was not seen until [time 2 – approximately 2.5 hours later] at which point was unwell **requiring admission to resus**.”

### Driver diagram

Our findings are mapped onto a driver diagram which shows the three main sources of unsafe care as the priority areas for change identified from these reports and potential improvement interventions (‘change ideas’) that would be suitable for evaluation (Fig. 2).

## Discussion

### Principal findings

Nine Coroners’ reports (from 1347 community and hospital reports, 2013–2018) and 217 NRLS reports (from 13 million, 2005–2015) were identified describing diagnostic errors with learning relevant to GP service provision in or alongside emergency departments. Initial theories include the following topics as potential priority areas for improvement interventions: inadequate triage and streaming processes; errors in clinical decision-making (under-investigation and mis-interpretation of results); and poor communication and referral pathways between the services. High risk presentations included musculoskeletal injury, chest pain, headache, calf pain and sick children.

### Strengths and limitations

There are recognised limitations in analysing these data. Coroners’ reports to prevent future deaths only cover the most severe of cases that have led to a patient death so the lessons derived from these cases may not be generalisable to cases with harm but not death outcomes. Analysis of NRLS patient safety incident reports is also limited by under-reporting, selection bias and incomplete analysis of causation [22, 36]. However, patient safety incident reports are recognised by the World Health Organization as ﻿useful to ﻿analyse the frequency of particular sources of harm, describe ﻿the nature of incident type and enable causes of harm and potential risks to be explored, supporting this mixed methods approach. Findings can then be used to inform local data collection and quality improvement projects to measure and monitor safe patient care in these settings [37, 38].

Diagnostic errors related to these service models may have been reported through other healthcare providers and therefore not identified through our NRLS filtering process. At the time of this study, NRLS reports were available up to 2015, updated searches may have identified further incident reports, potentially with additional themes indicating areas for improvement. GPs are recognised as low patient safety incident reporters, which may also have contributed to the low number of reports identified [22]. We have not included reports describing diagnostic errors occurring in usual emergency department care - whether GPs make relatively fewer or more errors on the same patients than emergency department staff doctors is unknown. The Cohen’s kappa statistic indicates that inter-rater reliability was moderate however this was strengthened by researcher one being an experienced GP and patient safety and qualitative researcher. Stakeholder feedback also strongly supported findings.

However, a strength is that many of the Coroners’ reports contained learning from in-depth root cause analysis, and which could be applied to near misses described in the NRLS reports. These two different lenses complement each other for understanding unsafe care, in terms of what happened and perceived causes for both the most serious and other incidents with a range of severity outcomes. Our small sample size is a limitation to the generalizability of our findings but the realist evaluation approach aims to build theory rather than provide definitive answers. Incorporating a realist approach into the thematic analysis of these data, we believe is novel. It structured the process of raising hypotheses to infer how and why these incidents occurred, and look for demi-regularities (patterns) to give greater understanding of possible causation. The *theories* can then be taken forward and tested against qualitative data collected/generated at case study sites with the aim of producing useful, insightful, actionable findings. These insights may also be applied to other new healthcare services where different healthcare providers take on additional roles. Systematic reviews may have identified further interventions to mitigate such events.

### Comparison with existing literature

Other studies support our findings that the causes of diagnostic errors in emergency departments are multifaceted, often with several contributory factors and have potential to result in serious patient harm [16, 39]. The Royal College of Emergency Medicine identified abdominal pain in the elderly, aortic dissection and cervical spine or hip fractures as the top three most common incident reports in emergency medicine following analysis of 61,449 incident reports in 2015 [40]. Our other work involved analysis of 2288 NRLS reports over a two year period (2013–2015) and described fracture (notably cervical spine and hip fractures), myocardial infarction and intracranial bleed as the most common diagnostic errors [25]. High-risk conditions for diagnostic errors described in community general practice do not include musculoskeletal injuries, headaches or veno-thrombotic events, [41] which may reflect the different cohort of patients (and ‘pre-test’ levels of risk) seen in these settings.

There is little national guidance on which patients should be streamed to GP services and this will depend on local service provision [42]. Initial NHS England guidance has adopted a model which advises against streaming patients with traumatic or head injuries and includes specific guidance for those presenting with chest pain, nosebleeds and feverish children [43]. There are however, established and internationally recognised triage systems which can help identify seriously ill patients who require urgent medical attention [44, 45]. Early warning scores for unwell children are available, [46] with more recent tools incorporating clinician ‘gut instinct’ and use by GPs [47]. The ‘Gestalt’ decision-making of senior nursing staff may also be better than algorithmic methods [48]. Our findings suggest that all patients who present to emergency departments, for the emergency or GP service, should be subject to a prompt standardised initial assessment, including basic observations.

The evidence for validated clinical decision-making tools in this setting is limited. Recognised tools to assess low risk chest pain include ECG and biochemical investigation results that may not be available to GPs working in emergency departments [49, 50] There are no validated risk assessment tools to assess patients presenting to the emergency department with headache, and the difficulty in identifying the few that do have a subarachnoid haemorrhage is acknowledged [51, 52]. Diagnostic cognitive processes and the effects of simplifying rules, short cuts or heuristics to replace more complex procedures are well described [53]. GPs that usually work in a lower risk community setting where there are often lower perceived and actual pre-test probabilities of serious disease may be at risk of errors in risk estimation. Cognitive debiasing strategies, [54] and reflective practice, [55] have been tried but their effectiveness in practice is questionable [56].

Poor communication and inadequate referral pathways between GP and specialist care are known to contribute towards patient safety incidents and can be targeted on a local level. Child safeguarding referral processes should be standardised between GP and emergency services [57]. Integrated computer systems including timely mandatory forcing functions for key information can improve communication [58, 59].

The lack of patient safety research in this area should be highlighted, [6, 20, 21] and teams should continue to learn from diagnostic errors, near misses and other patient safety incidents through local and national level reporting systems [60, 61].

### Implications for research and/or practice

Service providers and individual emergency departments will adopt a GP service model depending on their local circumstances and context [14]. Since this work was conducted, urgent and emergency care services along with almost all NHS service provision have changed due to the Covid-19 pandemic, including telephone screening of emergency department walk-in attendances, [62] and remote GP consultations [63]. Recommendations to improve future practice summarised in the driver diagram could however still be applied to these evolving healthcare service models. Implementation of the new Emergency Care Dataset (ECDS) in England, with the intent to extend soon into Ambulance and Integrated Urgent Care will ensure that in future there will be improved quantitative data to identify both presenting conditions and outcomes in patients who access Urgent and Emergency Care services to improve understanding of diagnostic errors in these settings. With better such data, the necessary evaluations of improvement interventions will be more feasible. These theories require refinement and testing with qualitative data collection from case study (hospital) sites.

## Conclusion

Initial theories include the following topics as potential priority areas for improvement interventions and evaluation to minimise the risk of diagnostic errors when GPs work in or alongside emergency departments: a standardised initial assessment with streaming guidance based on local service provision; clinical decision support for high-risk conditions; and standardised computer systems, communication and referral pathways between emergency and GP services.

## Data Availability

NRLS data is not publicly available due to the data sharing agreement at Cardiff University. The ‘Coroners reports to prevent future deaths’ dataset is available on the Courts and Tribunals Judiciary Website, https://www.judiciary.uk/related-offices-and-bodies/office-chief-coroner/https-www-judiciary-uk-subject-community-health-care-and-emergency-services-related-deaths/

## Abbreviations

GP: General Practitioner
ED: Emergency Department
